# On-Line Learning of Write Strategy for Ultra-Speed CD-RW Optical Recorder

**DOI:** 10.3390/s18072070

**Published:** 2018-06-28

**Authors:** Leehter Yao, June-Kai Huang

**Affiliations:** 1 Department of Electrical Engineering, National Taipei University of Technology, Taipei 10608, Taiwan; 2 Trend Rise Technology, Taichung 40842, Taiwan; ck.huang777@gmail.com

**Keywords:** infrared diode, write strategy, phase change media, CD-RW recorder, jitters, genetic algorithm, dynamic parameter encoding

## Abstract

An on-line machine learning approach integrating the genetic algorithm (GA) and jitter measurements is proposed to learn the write strategy for the infrared diode of ultra-speed CD-RW recorders. The recording performance differs significantly for the CD-RW discs recorded for the first, second, or third time above. It is difficult to learn one set of write strategy parameters for the infrared diode of ultra-speed CD-RW recorder that satisfies the recording specifications for three different types of discs. The GA is applied to the on-line learning of write strategy. However, the convergence of GA stagnates at the final stage of the learning process due to the fact that the write strategy parameters learned by the GA need to satisfy the recording specifications for discs recorded for the first time, second time and third time within one recording trial. To overcome this difficulty, a scheme called dynamic parameter encoding is proposed. This scheme improves the GA convergence and explores the search space much better than the conventional GA.

## 1. Introduction

The CD Rewritable (CD-RW) optical recorder provides functions of reading, writing, erasing, and overwriting information onto the optical disc. CD-RW recorders have become standard computer accessories due to their low cost and high data storage capacity. Phase-change recording media are mainly used for CD-RW recorders due to their attractive characteristics including high speed, high density, low power consumption, long life cycle and scalability [1,2,3]. Various write pulse generator circuits associated with the infrared diode generating the write pulse train (WPT) for both the CD-RW and DVD ± RW have been proposed. The WPT circuitry is controlled by a digital signal processor (DSP) in the recording servo control system. The control scheme implemented in the DSP fine-tunes the WPT, leading to well-formed pits and lands recorded on the disc. This is called the write strategy [4,5].

A good write strategy results in good recording performance, allowing the data recorded onto the optical disc to exactly follow the industry specifications [6,7]. The write strategy is implemented using a set of control parameters called write strategy parameters [6,7]. The write strategy parameters specify the laser power, pulse widths and delays of WPT generated from the infrared diode for the formulation of different lengths of pits and lands. Most of the write strategy parameters are correlated in terms of the effects on the recording performance. In other words, each parameter cannot be tuned independently. The tuning process conducted by experience engineers is laborious, time consuming and inaccurate, although it is currently the most widely adopted approach in the optical recording industry. Different disc manufacturers usually set different write strategies. A set of write strategy parameters corresponding to different types and brands of discs is designed and stored in the read-only memory of the DSP inside CD-RW recorder.

There have been some research articles investigating write strategies for optical recording devices. Phase change media features a large signal margin between its crystalline and amorphous states. The write pulse compensation on the pit length deviation was expressed using two simple linear equations [8]. With these two linear equations, a calibration approach was proposed to determine the write strategy after one-path test recording. One of the biggest challenges for optical recorder manufacturers is that some of the low-cost discs only marginally obey the standardized specifications, causing poor recording performance on these discs. To overcome this difficulty, jitter measurements and block error rates were both proposed in [9] to fine-tune the writing parameters instead of counting on the write strategy information pre-loaded by the manufacturer. The number of cycles is limited for repeated overwriting, especially if a fixed data pattern is used. A scheme based on random bit shifting was proposed in [10] to resolve this problem. Moreover, a high byte error rate exists when recording on high-speed rewritable optical discs. The cause of the high byte error rate is unwanted nucleation near the trailing edge of the amorphous marks. An effective scheme choosing proper cooling gap lengths in the write strategy was proposed in [11] to avoid this nucleation. The effects of different write strategies on the phase change memory have also been studied. The write strategy effects were analyzed through jitter measurements [12]. Different lengths of pits recorded on the optical disc according to different write strategies were compared and analyzed using atomic force microscopy [13]. The temperature rise on the phase change memory due to write strategy was also analyzed in [14]. New nanomaterials such as gold nanorods have the unique property of longitudinal surface plasmon resonance (LSPR) [15,16]. A 5-dimensional optical recording system including 3 spatial dimensions, wavelength and polarization was proposed in [17] utilizing the wavelength and polarization sensitivity of gold nanorods LSPR effects. A compact optical information storage sensing system on gold-nanorod-dispersed discs was designed in [18] utilizing a single diffractive optical element to separate the recording-reading laser beam from the servo beam. 

Developing an on-line auto-write strategy for optical recorders has been highly desired because it saves a lot of time and human effort to fine-tune the write strategy parameters. Different on-line calculation schemes have been proposed to determine the write strategy parameters by measuring pulse widths and RF signal levels [4,5]. However, the write strategy parameters learned by both on-line calculation schemes are not optimized. The write strategy specifications for regular speed CD-RW recorders [6] are less complicated than the ones for ultra-speed CD-RW recorders [7]. As the rotational speed is high, factors such as disc vibration, servo bandwidth and noise directly affect the recording performance [19]. In this paper, a novel automated on-line write strategy learning approach for the ultra-speed CD-RW recorder is proposed based on the genetic algorithm (GA) [20]. The automated write strategy learning approach aims to optimize the write strategy parameters for the ultra-speed CD-RW recorders. Different lengths of pit and land jitters are measured on-line and utilized as the indices for evaluating the recording performance associated with the write strategy under learning. The pit and land jitters are measured using the DSP of the CD-RW recorder, whereas the GA-based learning approach based on jitter measurements is implemented in a personal computer. The proposed novel approach coordinates both the firmware on the DSP of CD-RW recorder for jitter measurement and the software on a personal computer for optimal learning of write strategy parameters using GA.

A less complicated GA-based learning approach based on on-line measurements was applied to the optical dye recording device, CD Recordable (CD-R) in [21]. The write strategy learning approach proposed in this paper for phase change recording media is essentially different from the write strategy proposed for the dye recording media in [21], since phase change recording media have entirely differently characteristics from dye recording media. This leads to entirely different write strategy parameters for both recording media. Moreover, the recording performance of phase-change media differs significantly between discs that have been recorded for the first time, the second time, and the third time or more. The write strategy parameters learned for the discs recorded for first time cannot be applied to discs recorded for the second time or third time. On the other hand, the write strategy parameters learned for the discs that have been recorded 3 times or more do not work for discs to be recorded for the first time or the second time. The dye recording media do not have this recording uncertainty, and therefore the associated write strategy is much easier to design than the one for phase change recording media. In this paper, an approach learning one set of write strategy parameters that simultaneously applies to 3 different types of discs is proposed to solve this recording uncertainty for phase change media. Moreover, recording data onto the disc for the ultra-speed CD-RW drive tends not to be as accurate as for the regular or low-speed CD-RW drives. This is because it is technically difficult to fine-tune a laser beam applied to the phase-change disc and to create various lengths of pits and lands with high accuracy under high rotation speed. Therefore, it is difficult to further improve GA’s convergence at the final stage of the learning process because it is difficult to further fine-tune the write strategy parameters satisfying 3 different types of discs.

This convergence difficulty at the final stage of GA-based learning process also exists in other applications. A scheme called dynamic parameter (DPE) extension was proposed in [22] that adaptively controls the mapping from fixed-length binary genes to real values. DPE can further improve GA’s convergence by tracking the convergence of a population and using it to direct subsequent searches. A variant of DPE was proposed in [23] where a fuzzy inference system adaptively controlling the mapping from static binary genes to real-valued parameters was proposed. Both DPE [22] and its variant [23] change the parameterization resolution as mapping from the static binary genes to real values is adaptively changed. However, they cannot be directly applied to the GA-based write strategy learning approach proposed in this paper because the resolution needs to be fixed due to hardware limitations. A modified DPE with constant resolution (DPE_CR) is proposed in this paper. Along with the modified DPE_CR, a scheme similar to the operator called “extinction and immigration” is proposed in [24,25] to better improve GA’s convergence. The contributions of this paper are as follows:

(1) To our best knowledge, the proposed approach in this paper is the first automated design of write strategy for ultra-speed CD-RW recorder ever.
(2) An on-line learning approach is proposed that integrates the control of disc recording implemented on the CPU of CD-RW recorder and the high-performance computation of GA on the computer.
(3) An intelligent approach is proposed to further improve the convergence of GA for the learning of write strategy of CD-RW recorder with fixed memory resolution.
(4) The proposed learning approach is practically applied to the manufacturing of CD-RW recorders.

## 2. Configuration of CD-RW Recorder

**Figure 1:**
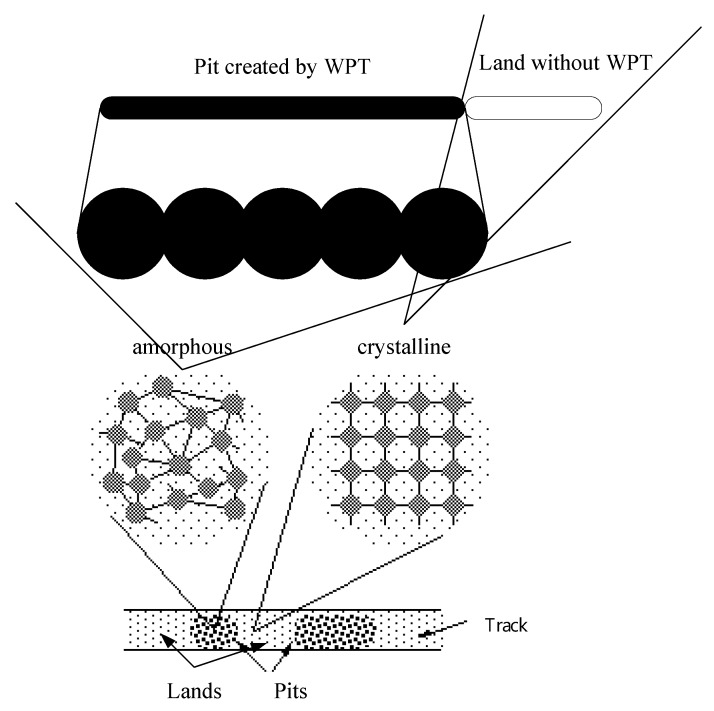
Formation of Pits and Lands on the disc recording layer.

Recording onto the CD-RW disc is based mainly on applying a write pulse train (WPT) from an infrared diode to the phase-change metal alloy layer on the disc. As shown in Figure 1, the material of the recording layer on the CD-RW disc is crystalline and has higher reflectivity to the laser for data reading. As the WPT generated by the infrared diode impinges on the disc, the material that the laser spot impinges on becomes amorphous and has decreased reflectivity. The mark (or pit) on the CD-RW disc is formed by a train of amorphous spots due to a WPT generated from the infrared diode, whereas the land is the part that remains crystalline. Every pit and land has a length ranging from 3*T* to 11*T*, where *T* is the length of one clock cycle.

**Figure 2:**
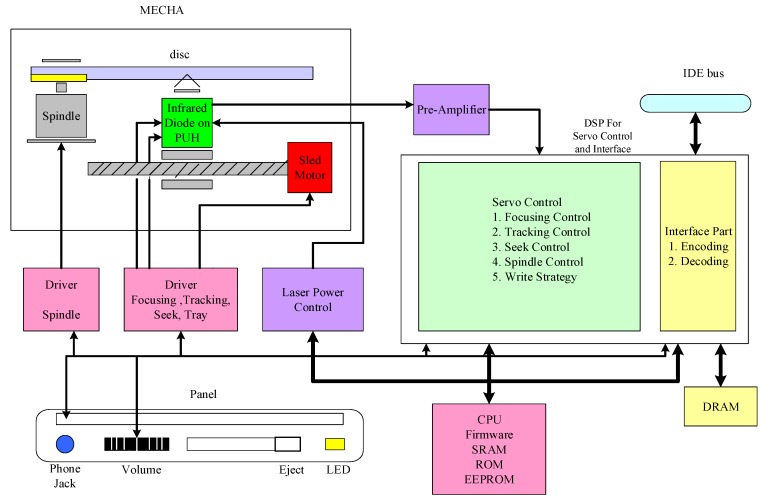
Configuration of CD-RW optical recorder.

To record the data processed at the computer onto the optical disc, the writing request and data are first transmitted through the IDE (Integrated Drive Electronics) bus to the digital signal processor (DSP) for servo control and interfacing as shown in Figure 2. The DSP first controls the spindle motor that rotates the disc. Based on the track location where the data is to be recorded on the disc, the DSP then controls the infrared diode on the pickup head (PUH) to inject a lower-power infrared signal reading tracks for track counting. Using the track counting signals, the DSP controls both the sled motor and PUH to perform track seeking. After the track where the data is to be recorded is located by the DSP’s track seek control, the DSP controls the infrared diode to inject a higher-power infrared WPT that changes the phase-change metal alloy layer on the disc from crystalline state to amorphous state forming different lengths of pits. As WPT is not applied to the disc, the original crystalline parts of the disc are formed as lands. While recording the data, the DSP also performs focusing and tracking control. Focusing control keep the PUH object lens away from the disc at a constant distance so that the recording laser from infrared diode can be focused onto the disc. Before being recorded onto the disc, the data sent from the computer are encoded using an eight to fourteen modulation (EFM) scheme on the DSP. A signal called the EFM signal based on the data encoded by the EFM scheme is generated as the reference signal for WPT control. Pits and lands with different lengths are formed by the WPT depending on the corresponding EFM signals. Tracking control keeps the PUH object lens following the disc track so that the laser from the infrared diode precisely follows the track while applying the WPT for data recording. The write strategy for the infrared red diode is designed and programmed in the DSP. According to the write strategy, different writing signals are sent to the infrared diode generating different WPTs. CD-RW also allows the data recorded on the disc to be erased. As the data erasing request sent from the computer through IDE bus to the DSP, DSP then controls the infrared diode to inject a lower-power infrared signal reading tracks to locate the file to be erased. After locating the file to be erased, a mediocre level of power is injected from the infrared diode, causing the sequence of pits associated with the file to be changed from the amorphous state to the crystalline state, i.e., changing associated pits to lands. 

## 3. Write Strategy for Optical Data Recording

The write strategies for CD-RW recorders with regular recording speeds such as 1X, 2X and 4X nominal CD speed are specified in [6], whereas the write strategies for CD-RW recorders with ultra-recording speeds such as 16X and 24X CD speeds are specified in [7]. Different write strategies are designed to fine-tune the WPT for different rotational speeds. The write strategies for the CD-RW recorder with regular recording speeds are generally simpler and easier to implement compared to the ones for CD-RW recorders with ultra-recording speeds.

**Figure 3:**
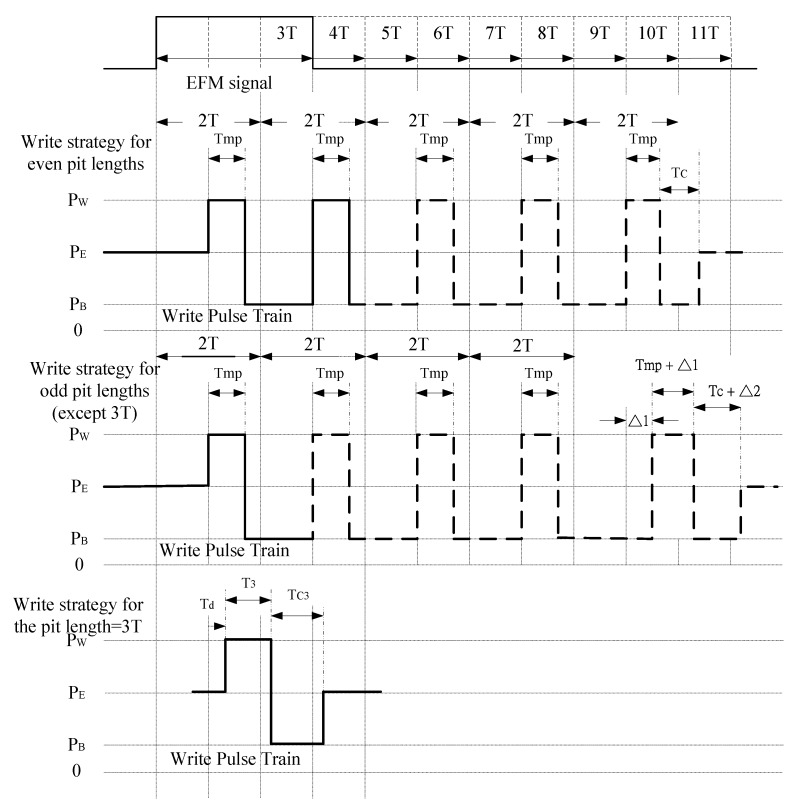
Write strategy for ultra-speed CD-RW recorder.

The write strategies for ultra-speed CD-RW recorders with 16X and 24X speeds are different from the ones for regular speeds. Without loss of generality, the write strategy for the CD-RW recorder with 24X speed is investigated in this paper. Since the disc is rotated at high speed for an ultra-speed CD-RW recorder, the number of write pulses in the WPT can be reduced compared to a regular speed CD-RW recorder. However, the write strategy is more complicated than the one for the regular speed CD-RW recorder. The write strategies for even and odd pit lengths are both specified in [7]. As shown in Figure 3, the pit with even and odd number of clock cycles *nT* is recorded by applying a WPT consisting of *n*/2 and (*n* − 1)/2 write pulses, respectively. Every write pulse but the last one of odd pit lengths has a length of $T_{mp}$ and repeats every 2*T*. In other words, every write pulse but the last one of odd pit lengths is (2*T* − $T_{mp}$) away from the trailing edge of previous pulse. For even pit lengths, the WPT is ended by a cooling interval allowing the power level to be kept as small as $P_{B}$ for a length of $T_{c}$ following the trailing edge of the last write pulse. When the WPT is ended, the laser power is set back to the level *P_E_*. For the odd pit lengths, the last write pulse has a length of ($T_{mp}$ + ∆1). The interval between the last write pulse and its previous one is also with an additional extension ∆1, which in total has a length of (2*T* − $T_{mp}$ + ∆1). The WPT is ended by a cooling interval with a length ($T_{c}$ + ∆2). As for a pit with 3*T* length, only one write pulse is required. The write strategy for a pit with 3*T* length is separately assigned. Referring to Figure 3, the write pulse has a length $T_{3}$ and starts at 1*T* of the EFM signal with a delay $T_{d}$. The cooling interval following the trailing edge of write pulse is $T_{c3}$. The parameters in the write strategy for ultra-speed CD-RW depicted in Figure 3 are summarized in Table 1. The write pulse width $T_{mp}$ is generally set as a constant, 7.23 ns [7]. Therefore, the write strategy is defined by a set of 6 parameters.

The write strategies for the ultra-speed CD-RW recorder are more difficult to implement than the ones for regular-speed CD-RW recorder. It is common for the engineers in the industry to manually tune the parameters by trial and error with the aid of experience and instrumentation measurements. Since the write strategy parameters need to be fine-tuned for all pits with different lengths, it usually requires a good deal of human effort to do so. In this paper, an automated learning scheme integrating both GA and on-line writing performance measurements is proposed to learn the write strategy parameters.

## 4. Evaluation of Write Strategies

Some of the parameters in the write strategy can be fine-tuned based on the recording performance. A recording performance index is required to evaluate the write strategy for a CD-RW recorder. Tuning the write strategy parameters directly affects the precision of both pits and lands. The expected length of the pits and lands ranges from 3*T* to 11*T*, where *T* = 231 ns. If the write strategy parameters are not correctly assigned, the pits and lands are not appropriately formed by WPT and the pit and land lengths differ from their expected values. Pit and land jitters are defined as the standard deviation of different pit and land recording lengths with respect to the expected values. Pit and land jitters are thus suitable for being utilized as the indices for evaluating recording performance corresponding to the write strategy. 

To evaluate the recording performance corresponding to different write strategy parameters, a set of pre-arranged computer data is utilized as the test data for recording. The size of test data used in this paper is 20 s long, containing 8.64 × 107 channel bits. Assume that $N_{kp}$ pits and $N_{kd}$ lands are in these 8.64 × 107 channel bits with expected length *kT*, *k* = 3…11. Let $L_{kp}^{i}$ and $L_{kd}^{i}$ be the *i*-th measured length of the pit and land, respectively, with expected length *kT*. Denote $J_{kp}$ and $J_{kd}$ as the jitter of pit and land, respectively; then

(1) $$J_{kp}^{}={\left(\frac{1}{N_{kp}}\sum_{i=1}^{N_{kp}}{\left(L_{{}_{kp}}^{i}-kT\right)}^{2}\right)}^{1/2},\;k=3\ldots 11.$$

(2) $$J_{kd}={\left(\frac{1}{N_{kd}}\sum_{i=1}^{N_{kd}}{\left(L_{{}_{kd}}^{i}-kT\right)}^{2}\right)}^{1/2},\;k=3\ldots 11.$$

The jitters $J_{kp}$ and $J_{kd}$ are utilized in this paper as the indices for evaluating the recording performance corresponding to the write strategy parameters. According to the industrial specifications in [7], both the pit and land jitters $J_{kp}$ and $J_{kd}$ are required to be less than 35 ns for *k* = 3…11. Please note that the jitter calculations are implemented based on (1) and (2) within the DSP.

**Figure 4:**
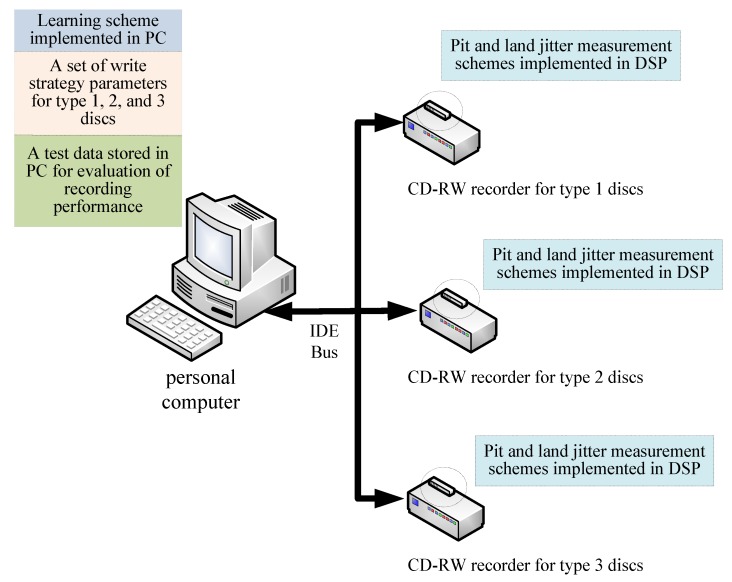
Learning one set of write strategy parameters satisfying 3 types of discs.

The recording performance of phase-change media differs significantly between discs that have been recorded for the first time, the second time, and the third time or more. A disc being recorded for the first time is referred to as a type 1 disc, and has never been recorded since it was manufactured. The disc recorded for the second time is referred as a type 2 disc, has only been recorded once, and the test data is to be overwritten on the same portion of the disc where the data were previously recorded. The disc recorded the third time or more is referred as a type 3 disc, and has been recorded at least twice. Generally, it is easier to fine-tune a set of write strategy parameters fulfilling the industrial specification, i.e., $J_{kp}$ and $J_{kd}$ less than 35 ns, for the disc being recorded for the first time. However, if the write strategy parameters are tuned aiming to fulfill the pit and land jitter specification simply for the type 1 disc, the recording performances are generally not satisfactory for type 2 and 3 discs. For instance, Table 2 shows pit and land jitters corresponding to a set of write strategy parameters tuned aiming to fulfill the specifications for type 1 discs. The pit and land jitters for different lengths are all less than 35 ns and fulfill the specifications for type 1 disc. However, if the same set of write strategy parameters is utilized in the same CD-RW recorder and the recording performance on type 2 and 3 discs was tested, the pit and land jitters are significantly greater than 35 ns, as also shown in Table 2. To resolve this uncertainty, a novel approach tuning the write strategy parameters individually to fulfill the pit and land jitters specifications for the 3 different types of discs is proposed, as in Figure 4. Figure 4 shows that 3 CD-RW optical recorders are connected by IDE bus with a personal computer recording data onto type 1, 2, and 3 discs, respectively. A GA-based learning scheme is designed in the personal computer (PC) to learn one set of write strategy parameters aiming to simultaneously satisfy the recording performance requirements for the 3 types of discs. A scheme calculating pit and land jitters based on (1) and (2) is implemented in the DSP inside each of the 3 CD-RW recorders in coordination with the GA-based learning scheme in the PC. The DSP sends back both pit and land jitters through the IDE bus to the computer in order for the GA-based write strategy learning approach to evaluate the recording performance. In order to evaluate and compare the recording performance of the write strategy under learning on 3 different types of discs, an identical set of 20-s-long data are recorded on these 3 different types of discs. The data source for these 3 CD-RW recorders to record on 3 different types of discs comes from the preset test data stored in the computer.

## 5. Learning of Write Strategy Parameters

The write strategy learning problem can be defined as an optimization problem. Let $\theta \equiv \left\{\theta_{1},\theta_{2},\ldots ,\theta_{6}\right\}$ be the set containing 6 write strategy parameters and θ∈Ξ where $\Xi \subset {\mathbb{R}}^{6}$ is the parameter space. An on-line approach M(θ) is required to evaluate the recording performance of CD-RW recorder based on the write strategy parameters θ under learning. The on-line recording performance evaluation approach M(⋅) is designed to measure pit and land jitters for 3 types of optical discs as shown in Figure 4. A cost function f(θ) associate the evaluation scheme M(θ) is designed to sum up the pit and land jitter measurements of 3 types of optical discs defined in (1) and (2) for the write strategy parameters θ. The optimal write strategy parameters $\theta^{*}$ is defined as the one minimizing the cost function as follows:

(3) $$\theta^{*}=\underset{\theta \in \Theta }{\arg\min}(f(\theta ))$$

It is obvious that an optimization approach O is required for the minimization in (3) in order to calculate the optimal write strategy parameters $\theta^{*}$. Since the cost function f(θ) is due to the on-line jitter measurements rather than a mathematical function, the minimization in (3) cannot be conducted through a mathematical optimization approach. Instead, a random optimization approach such as the GA is utilized as the optimization approach O in (3). The parameter space for the GA is defined in Ξ. It will be shown in this paper that the parametric search space for the GA in Ξ may change, while yet retaining a constant resolution due to hardware restrictions. Therefore, the write strategy learning for the ultra-speed CD-RW optical recorder is a 5-tuple optimization (θ,Ξ,M,f,O).

### 5.1. Genetic Algorithm and Fitness Function

The write strategy parameters are cascaded as a chromosome in the GA. Denote the number of bits utilized to encode every parameter in DSP as *ξ*. The chromosome is a binary string consisting of 6 cascaded *ξ*-bit binary sub-strings. For every pair of parent chromosomes, the cross-over operation is implemented parameter by parameter. One splice point for cross-over operation is randomly selected among the *ξ*-bit substring corresponding to the parameter. The write strategy parameters encoded in every chromosome determine the WPT patterns, which lead to the formation of pits of different lengths. A pit on the CD-RW disc is made using a train of amorphous spots due to the WPT. The land adjacent to the pit is the crystalline part not recorded by WPT. Although write strategy parameters lead to pit formation, the land formation is also affected by the write strategy parameters because pits and lands are adjacent to each other on the same optical track. As described in the previous section, the pit and land jitters shown in (1) and (2), respectively, are utilized to evaluate the recording performance due to the write strategy parameters encoded in every chromosome. However, since the recording performance differs significantly for 3 types of discs, the write strategy parameters learned by GA need to have satisfactory writing performance on 3 different types of discs at the same time. Similar to the pit and land jitters in (1) and (2), denote $J_{kp}^{\gamma }$ and $J_{kd}^{\gamma }$, *k =* 3…11, as the pit and land jitters for the type *γ* disc, *γ* = 1, 2 and 3. Therefore, every set of write strategy parameters encoded in a chromosome is applied to three different CD-RW recorders at the same time, evaluating the recording performance on 3 different types of discs. The pit and land jitters $J_{kp}^{\gamma }$ and $J_{kd}^{\gamma }$, *k* = 3…11, *γ* = 1, 2, and 3, are measured individually on the 3 different types of discs as the fitness value for the chromosome.

Since both the pit and land jitters are required to be less than 35 ns, pit and land jitters greater than 35 ns are penalized in the fitness function to increase GA’s convergence rate. Let *Θ* (*i*,*g*) be the set of write strategy parameters encoded in the *i*-th chromosome of the *g*-th generation. Since there are 6 write strategy parameters to be learned, $\Theta (i,g)=\{\theta_{1}(i,g),\theta_{2}(i,g),\ldots ,\theta_{6}(i,g)\}$, where $\theta_{j}(i,g)$, *j* = 1…6, are the parameters defined in Table 1.

Denote the weighted pit and land jitters corresponding *Θ* (*i*,*g*) as $\Lambda_{kp}^{\gamma }(i,g)$ and $\Lambda_{kd}^{\gamma }(i,g)$, then,

(4) $$\Lambda_{kp}^{\gamma }(i,g)=\{\begin{array}{c} J_{kp}^{\gamma }(i,g),\;\mathrm{if}\;J_{kp}^{\gamma }(i,g)\leq \;35\;\mathrm{ns},\;\\ \eta \times J_{kp}^{\gamma }(i,g),\;\mathrm{if}\;J_{kp}^{\gamma }(i,g)>\;35\;\mathrm{ns}; \end{array}$$

(5) $$\Lambda_{kd}^{\gamma }(i,g)=\{\begin{array}{c} J_{kd}^{\gamma }(i,g),\;\mathrm{if}\;J_{kd}^{\gamma }(i,g)\leq \;35\;\mathrm{ns},\;\\ \eta \times J_{kd}^{\gamma }(i,g),\;\mathrm{if}\;J_{kd}^{\gamma }(i,g)>\;35\;\mathrm{ns}; \end{array}$$

∀*k* = 3…11, *γ* = 1…3.

Please note that a penalty factor *η >* 1 is assigned in (4) and (5) to jitters greater than 35 ns. Let *f*(*i*,*g*) be the fitness function associated with the *i*-th chromosome in the *g*-th generation, *f*(*i*,*g*) is define as the sum of different lengths of pit and land jitters on all 3 types of discs, i.e.,

(6) $$f(i,g)=\sum_{\gamma =1}^{3}\sum_{k=3}^{11}(\Lambda_{kp}^{\gamma }(i,g)+\Lambda_{kd}^{\gamma }(i,g))$$

Assume that *N* chromosomes are assigned in the gene pool. The best set of write strategy parameters learned in the *g*-th generation denoted as $\Theta^{*}\left(g\right)\equiv$$\left\{\theta_{1}^{*}\left(g\right),\theta_{2}^{*}\left(g\right),\ldots ,\theta_{6}^{*}\left(g\right)\right\}$ corresponds to the minimum fitness value among all *N* chromosomes in the gene pool, i.e.,

(7) $$\Theta *(g)=\underset{i=1\ldots N}{Argmin}(f(i,g))$$

The best fitness value in the *g*-th generation denoted as f*(*g*) is defined as the minimum fitness value corresponding to $\Theta^{*}\left(g\right)$. The elitist approach, i.e., the approach passing the chromosome with the least fitness value in the entire gene pool to the next generation, is utilized in the chromosome reproduction.

### 5.2. Extended Parameter Encoding with Constant Resolution

Referring to (6) and (7), the write strategy parameters decoded from every chromosome needs to satisfy the pit and land jitter specifications for all 3 types of discs. Practically, it is difficult for the GA to learn the near optimum parameters satisfying the jitter specifications for 3 different types of discs at the same time. Although GA is capable of learning the parameters that lead to decreasing convergence for the fitness values, GA’s convergence usually stagnates as the parameters under learning are close to the optimal solutions. The difficulty mainly comes from the fact that it is practically difficult to search a set of write strategy parameters that is better suited to all 3 types of discs at the same time when the parameters are close to the optimal solution. To overcome this convergence stagnation at GA’s final stage of convergence, a novel encoding approach called Dynamic Parameter Encoding with Constant Resolution (DPE_CR) is proposed. The DPE_CR is applied when the GA with regular intermediate mapping returns the same best fitness value f*(·) for *α*_1_ consecutive generations. The basic idea of DPE_CR is to focus GA’s search space around the solution that GA has converged for a certain number of generations.

As DPE_CR is applied, the parameters under learning are close to the optimal solution. The number of chromosomes in the gene pool is further reduced to ease the computational efforts. Assume that *Q* bits instead of the original *L* bits are utilized to encode the parameters for DPE_CR. *Q* is set to be significantly less than *L*. As the number of bits is reduced from *L* to *Q*, the decoding of every chromosome needs to be redefined. The converged gene pool is also reset after DPE_CR is applied. As a new encoding approach is applied to every chromosome, it can be considered that a new “era” has been brought to the gene pool. Let the GA with the original encoding approach be the first era and the GA with DPE_CR being applied for the *z*-th time be the (*z* + 1)-th era.

An intermediate mapping rather than a direct decoding from binary string is designed to work along with DPE_CR, decoding the binary string associated with every parameter. The range of each write strategy parameter for the CD-RW recorder with 24X speed is shown in Table 3. Denote $\theta_{j}^{z}(i,g)$ as the decoded *j*-th write strategy parameter contained in the *i*-th chromosome of *g*-th generation in the *z*-th era. Let [$v_{1j}^{z}$, $v_{2j}^{z}$], *j* = 1…6, be the range of variation for $\theta_{j}^{z}(i,g)$. Recall that ξ bits are utilized to encode $\theta_{j}^{z}(i,g)$, and denote $b_{j}^{z}(i,g)$ as the associated binary string. The intermediate mapping for every write strategy parameter $\theta_{j}^{z}(i,g)$ is a one-to-one mapping from the binary representation to the real value within the range of variation, i.e.,

(8) $$\theta_{j}^{z}(i,g)=\frac{b_{j}^{z}(i,g)}{2^{\xi }-1}(v_{2j}^{z}-v_{1j}^{z})+v_{1j}^{z},\;\forall \;j=1\ldots 6.$$

For the regular GA without DPE_CR, the parameter encoding is not changed as GA has converged close to the optimum. It is difficult for the GA to further improve the convergence, simply relying on cross-over and mutation operations on the *L*-bit binary representation of every parameter with intermediate mapping to the entire search space. DPE_CR reduces the search space to a smaller area around the converged solutions. In other words, the upper and lower boundary of the search space for intermediate mapping is appropriately shrunk toward the previous converged solution obtained before the DPE_CR is applied, using fewer numbers of bits to encode every parameter. Reducing the number of bits to encode the parameter for intermediate mapping allows GA’s parameter search to be more focused and efficient. It is worth noting that although DPE_CR reduces both the search space for intermediate mapping and the number of bits utilized for parameter encoding, the parameter resolution is kept the same for the sake of hardware implementation.

Referring to Table 3, the maximum range of variation for every write strategy parameter ranges from 0.5*T* to 1.5*T*. Recall that *T* is the length of one clock cycle, which is 231 ns/bit. With such a small range of variation, extra care of resolution adapting with hardware limitations is required, as an *L*-bit substring is assigned to encode every write strategy parameter. The laser LED on the PUH is controlled to generate the WPT according to the write strategy parameters. Ideally, every write strategy parameter can be precisely assigned a value with good resolution. However, due to cost considerations and hardware limitations on the control of laser LED, the resolution of write strategy parameters is set as a reasonable value. Even though the dynamic parameter encoding approach allows GA to zoom in the search space around the local minimum, the resolution needs to be kept the same. This is different from the DPE proposed in [22,23]. The control of laser LED on PUH cannot be delicately achieved with extremely high resolution. Referring to (8), the parameter resolution $\delta_{j}$ with or without DPE_CR for the *j*-th parameter in the chromosome is defined as

(9) $$\delta_{j}=\frac{v_{2j}^{1}-v_{1j}^{1}}{2^{L}-1}$$

where $v_{1j}^{1}$ and $v_{2j}^{2}$, are given as the maximum range specified in Table 3.

With the resolution $\delta_{j}$ being kept the same, the upper bound and lower bound of variation range for the *z*-th era and *z* ≥ 2, denoted as $v_{2j}^{z}$ and $v_{1j}^{z}$, respectively, are defined as follows:

(10) $$v_{2j}^{z}=(\theta_{j}^{z-1})*+(2^{\xi -1}-1)\delta_{j}$$

(11) $$v_{1j}^{z}=(\theta_{j}^{z-1})*-2^{\xi -1}\delta_{j}$$

where ${\left(\theta_{j}^{z-1}\right)}^{*}$ denotes the *j*-th best parameter obtained in the previous (*z* − 1)-th era. Let the generation in which the *z*-th era starts be *G_z_*, then

(12) $${\left(\theta_{j}^{z}\right)}^{*}\equiv \theta_{j}^{*}(G_{z}-1)$$

Denote the best fitness value obtained in the *z*-th era where $f_{z}^{*}$, $f_{z}^{*}$ is the fitness value associated with ${\left(\theta_{j}^{z}\right)}^{*}$, i.e.,

(13) $${\left(f_{z}\right)}^{*}\;\equiv f^{*}\left(G_{z}-1\right)$$

Referring to (10)–(12), the parameters obtained from the previous era are set as the center of the new search space in the current era. The resolution $\delta_{j}$ remains unchanged through GA’s entire learning process. The GA with DPE_CR as in (10)–(12) is run until GA’s best fitness value has not changed for $\alpha_{2}$ generations. Recall that the condition under which the first DPE_CR is started is set as when the best fitness value $f^{*}\left(\cdot \right)$ has not changed for $\alpha_{1}$ generations. Since the parameters under learning in the first era are implemented using a longer binary sub-string, the variation range is wider than the range for the parameters in subsequent eras. The learning saturation tolerance in the first era is designed to be less strict than the one for the following eras so that the learning process in the first era can evolve for more generations and achieve learning results closer to the optimal solution before the first DPE_CR is applied. The number of generations $\alpha_{1}$ for which allows $f^{*}\left(\cdot \right)$ to be unchanged before the application of DPE_CR is designed to be larger than $\alpha_{2}$.

To prevent the GA from getting trapped at a steep local minimum, making it difficult to improve the convergence further when the search space is not large enough, a mechanism expanding the search space is designed. As soon as the DPE_CR is applied, the search space is doubled. In other words, ξ is increased by 1. Referring to (10) and (11), if ξ is updated as (ξ + 1), the upper and lower limit of the search space is doubled with constant parameter resolution. As long as DPE_CR leads to no convergence, the search space expansion mechanism continues until ξ is increased to an upper limit (*L* − 1) that stands for half of the entire GA search space. The stop criteria of GA are met if the fitness values have not been improved for $G_{s}$ generations or the parameter under learning leads to the condition that both the pit and land jitters are less than 35 ns for the 3 types of discs. Regardless how large ξ has been increased to, ξ is reset to *Q* as long as DPE_CR leads to fitness value improvement. In other words, as long as the parameter under learning leads to further fitness value improvement, the GA search space is reduced to the neighborhood around the current best solution, allowing a more focused search. Therefore, the value of ξ is updated in every era as follows:

(14) $$\xi =\{\begin{array}{c} \begin{array}{c} L,\;\;\;\;\;\;\;\;\;\;\;\;\;\mathrm{if}\;z\;=\;1;\\ Q,\;\;\;\;\;\;\;\;\;\;\;\;\;\mathrm{if}\;z\;=\;2; \end{array}\\ \xi +1,\;\mathrm{if}\;z\geq 3\;\mathrm{and}\;f_{z}^{*}=f_{z-1}^{*};\\ Q,\;\mathrm{if}\;z\geq 3\;\mathrm{and}\;f_{z}^{*}<f_{z-1}^{*}. \end{array}$$

Please note that ξ is updated according to (14) under the condition that ξ ≤ (*L* − 1).

### 5.3. Coordination between GA-Based Learning and On-Line Jitter Measurement

Because the GA requires intensive computation and large computer memory, the learning of write strategy parameters through the GA is implemented in a PC instead of the DSP of the CD-RW recorder, as shown in Figure 2. However, referring to (6), the fitness value of every chromosome in the GA relies on on-line pit and land jitter measurements from 3 types of discs at the same time. The PC is designed to install 3 CD-RW recorders running these 3 different types of disc. The jitter measurement is implemented on the DSP of every CD-RW recorder. A coordination scheme between the PC and DSP of every CD-RW recorder is designed to work along with the GA-based learning scheme so that the hardware measurement results made by the DSP of the 3 CD-RW recorders can be efficiently utilized as the fitness values for the software computations conducted in the PC.

To calculate the fitness value of every chromosome, the write strategy parameters decoded from the chromosome are sent to the DSP of every connected CD-RW recorder through the IDE bus. Every CD-RW recorder is controlled to record a 20-s-long benchmark test data based on the received write strategy parameters. The DSP of every CD-RW recorder is designed to measure the pit and land jitters and send the results back to the PC through the IDE bus. Upon receiving the pit and land jitters from the 3 CD-RW recorders, the PC processes the jitters by assigning appropriate weights according to (4) and (5) and then sums the weighted jitters as the fitness value in (6). The coordination between the GA-based learning scheme on the PC side and the jitter measurements on the CD-RW recorder side is described as follows.

GA-based Learning on PC Side

Step 0: Set *g* = 0, *c* = 0 and *z* = 1. Set the fitness value tolerance $\tau_{s}$ and generation tolerance without improvement $G_{s}$. Set the generation tolerance $\alpha_{1}$ to start the second era and $\alpha_{2}$ to trigger the 3rd era or above.
Step 1: Set $v_{1j}^{1}$ and $v_{2j}^{1}$, *j* = 1…6, according to the specifications list in Table 3. Calculate the resolution $\delta_{j}$ based on (9).
Step 2: Determine ξ based on (14).
Step 3: If *z* ≥ 2, calculate $v_{1j}^{z}$ and $v_{2j}^{z}$, *j* = 1…6, according to (10) and (11), respectively. Initialize the gene pool.
Step 4: *g* = *g* + 1. For *i* = 1 to *N*, repeat steps 5 to 14.
Step 5: Calculate $\theta_{j}^{z}(i,g)$, *j* = 1…6, according to the intermediate mapping in (8).
Step 6: For *γ* = 1 to 3, repeat steps 7 to 11.
Step 7: Send $\theta_{j}^{z}(i,g)$, *j* = 1…6, through IDE bus to the DSP in the *γ*-th CD-RW recorder.
Step 8: Command the *γ*-th CD-RW recorder to record a pre-stored 20-s long benchmark test data based on the received write strategy parameters.
Step 9: Start the pit and land jitter measuring process at the CD-RW recorder side as Steps 9.1 to 9.7.
Step 10: Check if the “Change Disc” request from the γ-th CD-RW recorder is received and the disc tray is open. If yes, go to step 11; otherwise go to step 12.
Step 11: Change a new blank disc for recording. Close the disc tray.
Step 12: While data transmission request is on from the CD-RW recorder, receive the measured jitters for different lengths of pits and lands from the γ-th CD-RW recorder, $J_{kp}^{\gamma }(i,g)$ and $J_{kd}^{\gamma }(i,g)$, *k* = 3…11, γ = 1…3, through the IDE bus.
Step 13: Calculate weighted pit and land jitters, $\Lambda_{kp}^{\gamma }(i,g)$ and $\Lambda_{kd}^{\gamma }(i,g)$, *k* = 3…11, *γ* = 1…3, according to (4) and (5), respectively.
Step 14: Calculate the fitness value *f*(*i*,*g*) of the *i*-th chromosome in the *g*-th generation.
Step 15: Rank the fitness values associated with the chromosomes in the gene pool.
Step 16: Determine the least fitness value $f^{*}\left(g\right)$. Pass the best chromosome with $f^{*}\left(g\right)$ into the (*g* + 1)-th generation. Apply gene selection and crossover operations to generate the rest of (*N* − 1) chromosomes.
Step 17: If the best fitness value of the *g*-th generation $f^{*}\left(g\right)\leq \tau_{s}$ or *c* ≥ $G_{s}$, stop the algorithm; otherwise go to Step 18.
Step 18: If the best fitness value $f^{*}\left(g\right)=f^{*}\left(g-1\right)$, *c* = *c* + 1; else *c* = 0.
Step 19: If (*z* = 1) and (*c* = $\alpha_{1}$), go to Step 20; otherwise if (*z* ≥ 2) and (*c* = $\alpha_{2}$), go to Step 20; otherwise go back to step 4.
Step 20: ${\left(\theta_{j}^{z}\right)}^{*}=\theta_{j}^{*}(g)$, *j* = 1…6. $f_{z}^{*}=f^{*}\left(g\right)$.
Step 21: *z* = *z* + 1. If *z* ≥ (*L* − 1), *z* = *L* − 1.
Step 22: go back to step 2.

Jitters Measuring Process at the *γ*-th CD-RW Recorder Side:

Step 9.1: Receive the writing parameters $\theta_{j}\left(i,g\right)$, *j* = 1…6, from the PC side through IDE bus.
Step 9.2: Check if the disc is full for recording. If yes, go to step 3, otherwise go to step 5.
Step 9.3: Signal a “Change Disc” request through IDE bus to the PC. Open the disc tray.
Step 9.4: Transform the write strategy parameters into corresponding WPT for the data recording.
Step 9.5: Record 20 s of pre-determined benchmark test data into the disc based on the corresponding WPT. The pre-determined benchmark data are retrieved from the PC through the IDE bus.
Step 9.6: Measure the jitters for different lengths of pits and lands, $J_{kp}^{\gamma }(i,g)$ and $J_{kd}^{\gamma }(i,g)$, *k* = 3…11.
Step 9.7: If recording process is completed, transmit the jitters $J_{kp}^{\gamma }(i,g)$ and $J_{kd}^{\gamma }(i,g)$, *k* = 3…11, to the PC through IDE bus.

## 6. Experiments

The experiments for the proposed write strategy learning approach are conducted on CD-RW recorders with 24X speed. A PC installed with 3 CD-RW recorders is utilized as the experiment platform. The fitness tolerance $\tau_{s}$ for GA’s stopping criteria is set as 1728 based on the assumption that the average pit and land jitters for every type of disc is 32 ns. Therefore, 32 ns × 9 × 2 × 3 = 1728 ns. Another stopping criterion, the generation tolerance $G_{s}$ for fitness without improvement is set as 50. The generation tolerances to start the second era and the third era or above, i.e., $\alpha_{1}$ and $\alpha_{2}$, are to be 25 and 10, respectively. The gene pool of GA is designed to contain 30 chromosomes. Since the DSP of every CD-RW recorder utilizes 1 byte to implement every write strategy parameter, the number of bits *L* in the first era in (14) is set as 8. The reduced size of bits for DPE, i.e., *Q* in (14), is set as 5. The penalty factor η in (4) and (5) is set to 20 for penalizing jitters greater than 35 ns.

Referring to (6), the fitness value is defined as the summation of jitter measurements from 3 CD-RW recorders. As the fitness value is less than the fitness tolerance $\tau_{s}$, the learning results are good enough to fit the preset threshold, and the GA is stopped because the stopping criteria are triggered. The stopping criteria for the total jitter measurements have been preset and the stopping criterion for each individual CD-RW is also set. The recording performance of every CD-RW recorder in response to the same write strategy parameters is different. As long as the recording performance made by any of the CD-RW satisfies the stopping criterion, the most time-consuming part of the jitter measurement can be stopped, saving computational efforts. The stopping criterion for every individual CD-RW recorder is set as 1/3 of $\tau_{s}$, which is 576 ns. As long as any of CD-RW recorders results in accumulated pit and land jitter measurements of less than 576 ns, it stops measuring the pit and land jitters of the 20-s test data and returns the final jitter measurement as the fitness values for the following generations until the GA is terminated.

**Figure 5:**
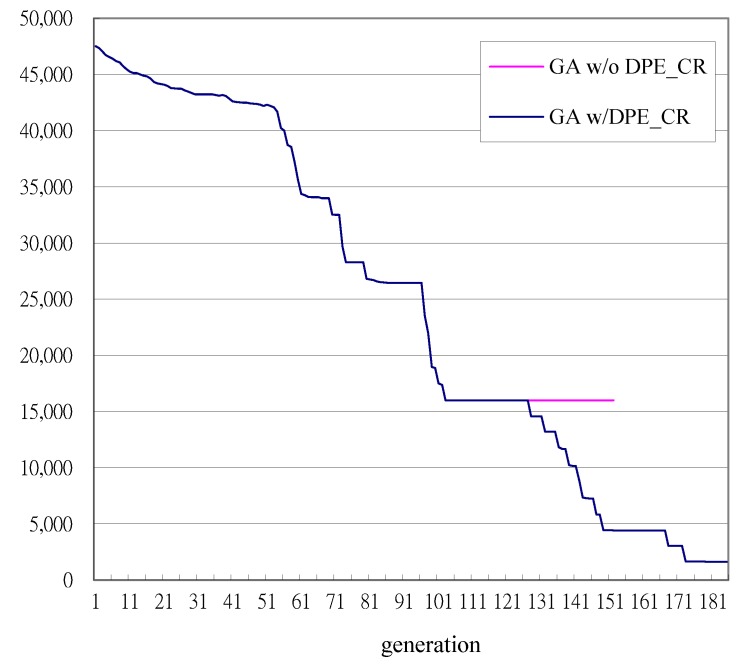
Convergence of fitness values for the GA with DPE_CR and without DPE_CR.

**Figure 6:**
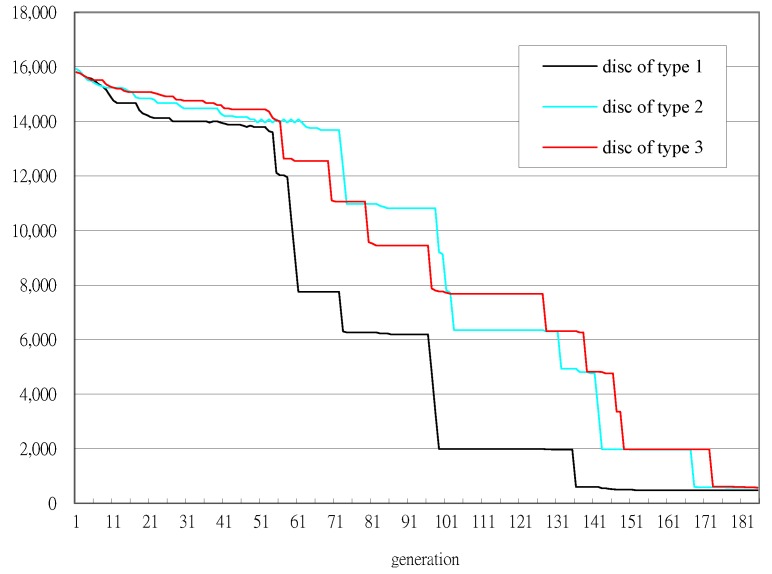
Convergence of the fitness values measured from every individual CD-RW recorder.

The convergence of GA with DPE_CR is shown in Figure 5. There are 3 eras in the entire GA learning process. The first era ends at the 127th generation since the fitness value 12,006 has remained the same for $\alpha_{1}$ (=25) generations. As soon as the first era ends, the DPE_CR is applied, with the number of bits utilized to encode every write strategy parameter being reduced from *L* (=8) bits to *Q* (=5) bits. Figure 5 shows that DPE_CR successfully improves GA’s convergence because DPE_CR helps focus the search space around the solutions that GA has achieved in the first era. The fitness values are further reduced starting from the 127th generation and continuing until the 158-th generation, where the fitness values stop improving. After waiting for $\alpha_{2}$ (=10) generations of no fitness value improvements, the third era begins at the 168th generation and the number of bits to encode every write strategy parameter is increased from 5 bits to 6 bits. It is shown in Figure 5 that GA’s convergence is further improved by the DPE_CR initiated in the third era. It is worth noting that the first CD-RW recorder returns a jitter measurement less than 576 ns at the 168th generation, while the second CD-RW recorder does so at the 177th generation before the GA is terminated at the 185th generation. Therefore, the first and second CD-RW recorders stop measuring jitters at the 168th and 177th generation, respectively. However, the jitters measured at the 168th generation for the first CD-RW recorder and the jitters measured at the 177th generation for the second CD-RW recorder are taken as the fitness values for the remaining generations until the final 185th generations. The convergences of fitness values for every CD-RW recorder recording the disc of type 1, 2 and 3, respectively, are shown in Figure 6.

To show the effectiveness of DPE_CR, the recording performances based on the write strategy parameters learned by the GA with and without DPE_CR are also compared in Figure 5. Referring to Figure 5, the first DPE_CR is applied at the 127th generation, where the first era ends and the second era begins. In order to have a fair comparison, all of the chromosomes in the gene pool at the 127th generation evolved by the GA with DPE_CR are saved and continue to evolve but without DPE_CR. Figure 5 shows that the convergence stagnates if the GA is applied without DPE_CR. This is because the regular GA without DPE_CR finds it difficult to evolve the write strategy parameters learned up to the 127th generation to further reduce the pit and land jitters for 3 types of discs in a limited number of generations. Recall that the generation tolerance *G_s_* for fitness without improvement is set as 50, the convergence of the GA without DPE_CR is stopped as the stopping criterion is met.

To show the effectiveness of the proposed GA with DPE_CR, the pit and land jitters due to the write strategy determined by the GA with DPE_CR are compared with the jitters measured from a typical CD-RW recorder purchased from the market. Comparisons are made in Table 4, Table 5 and Table 6 for the discs of type1, 2 and 3, respectively. The conventional approach shown in Table 4, Table 5 and Table 6 refers to the trial and error approach based on engineers’ experience and instrumentation measurements. The conventional approach is usually adopted in a typical CD-RW recorder purchased from the market. The proposed GA with DPE_CR was also run 10 times, learning the write strategy parameters under the same stopping criteria in order to test the reliability of the proposed modified GA. It is shown in Table 7 that the write strategy parameters were successfully obtained for 3 types of discs with different number of generations.

Since the PC is connected to 3 CD-RW recorders, recording 20-s benchmark test data on every CD-RW recorder is not conducted in sequence. To save fitness value evaluation time, the jitter measurements for the discs in 3 different CD-RW recorders are conducted in parallel. It takes 25.8 s on average for the PC to evaluate the fitness value associated with every chromosome. It takes 143,278 s (≈39.8 h) to produce the convergence profile shown in Figure 5 on a PC with a 3.6 GHz CPU. This especially signifies the efficiency of the proposed learning approach based on the GA with DPE_CR, because it usually takes at least 10 working days for a group of 3 experienced engineers to fine-tune a set of workable write strategy parameters. The GA-based learning approach takes much less time to find the write strategy parameters. Moreover, the solution obtained by the GA-based learning approach leads to smaller pit and land jitters, as shown in Table 4, Table 5 and Table 6.

## 7. Conclusions

An on-line automated write strategy learning approach for the infrared diode of ultra-speed CD-RW recorders has been proposed. This method successfully found the write strategy parameters, leading to good recording performance. The idea of integrating write strategy parameter learning on the PC side with the jitter measurements on the optical recorder side can also be applied to other phase-change recording media. The proposed learning approach leads to less human effort and better recording performance. The proposed idea is especially suitable for write strategy design in the optical recorder industry because research engineers are required to design the write strategy for every type and brand of optical disc. The proposed learning approach can greatly save human labor and yet maintain recording quality. Moreover, the response time to write strategy design request for new types or brands of optical discs from customers can be greatly reduced for optical recorder manufacturing companies.

## Figures and Tables

**Table 1:** Parameters implemented in the write strategy for the ultra-speed CD-RW recorder.

Parameter	Descriptions
$T_{mp}$	write pulse width for odd and even pit lengths. (It is generally set as a constant 7.23 ns.)
∆1	additional extension of the interval between the last write pulse and its previous one for odd pit lengths.
∆2	additional extension of the cooling interval for odd pit lengths.
$T_{c}$	cooling interval for both odd and even pit lengths.
$T_{d}$	additional delay for the pit length = 3*T*.
$T_{3}$	write pulse width for the pit length = 3*T*.
$T_{c3}$	cooling interval for the pit length = 3*T*.

**Table 2:** Comparison of pit and land jitters for the disc recorded first, second and third time or above.

Length	Disc Recorded First Time		Disc Recorded Second Time		Disc Recorded Third Time or Above	
	Pit Jitters	Land Jitters	Pit Jitters	Land Jitters	Pit Jitters	Land Jitters
3*T*	32.14	33.65	55.74	39.96	49.74	32.09
4*T*	30.62	31.36	52.31	53.47	46.53	47.53
5*T*	30.53	30.03	52.87	53.13	46.74	47.62
6*T*	30.36	29.59	54.71	54.27	49.16	49.68
7*T*	29.02	29.42	55.98	55.33	50.61	51.47
8*T*	29.04	29.32	56.49	56.05	52.34	52.90
9*T*	28.98	29.79	55.87	57.78	51.16	53.40
10*T*	29.73	29.60	58.30	59.87	53.91	55.20
11*T*	28.02	29.90	52.76	53.99	48.29	49.85

**Table 3:** Range of every Write Strategy Parameter for CD-RW recorders with 24X nominal speed.

Write Strategy Parameter (Notation)	For 24X Nominal Speed
$T_{mp}$	7.23 ns (fixed)
$\Delta_{1}\left(\theta_{1}\right)$	0*T*–0.5*T*
$\Delta_{2}\left(\theta_{2}\right)$	0*T*–0.5*T*
$T_{c}\left(\theta_{3}\right)$	0*T*–1.0*T*
$T_{d}\left(\theta_{4}\right)$	−0.5*T*–0.5*T*
$T_{3}\left(\theta_{5}\right)$	1.0*T*–2.5*T*
$T_{c3}\left(\theta_{6}\right)$	0.25*T*–1.0*T*

**Table 4:** Comparison of jitters by GA with DPE_CR and conventional approach for the disc of type 1.

Length	Pit Jitters (ns)		Land Jitters (ns)	
	GA w/DPE_CR	Conventional Approach	GA w/DPE_CR	Conventional Approach
3*T*	27.26	32.49	29.21	33.14
4*T*	27.19	32.11	27.50	33.63
5*T*	27.16	33.01	26.82	33.07
6*T*	27.01	32.54	27.45	32.44
7*T*	26.63	32.76	26.21	31.64
8*T*	26.99	33.26	26.31	32.54
9*T*	26.58	31.51	25.90	32.41
10*T*	26.58	31.57	26.04	32.48
11*T*	26.03	32.20	27.25	33.23
average	26.83	32.38	26.97	32.73

**Table 5:** Comparison of jitters by GA with DPE_CR and conventional approach for the disc of type 2.

Length	Pit Jitters (ns)		Land Jitters (ns)	
	GA w/DPE_CR	Conventional Approach	GA w/DPE_CR	Conventional Approach
3*T*	30.47	34.89	33.48	34.49
4*T*	30.07	34.43	31.91	33.87
5*T*	28.38	34.87	31.67	33.25
6*T*	30.89	33.89	32.21	33.91
7*T*	32.26	34.16	31.78	32.98
8*T*	32.37	33.78	31.52	32.25
9*T*	32.45	33.25	30.96	33.56
10*T*	32.66	33.14	31.43	33.78
11*T*	32.89	34.87	31.77	34.88
average	31.38	34.14	31.86	33.66

**Table 6:** Comparison of jitters by GA with DPE_CR and conventional approach for the disc of type 3.

Length	Pit Jitters (ns)		Land Jitters (ns)	
	GA w/DPE_CR	Conventional Approach	GA w/DPE_CR	Conventional Approach
3*T*	31.46	33.49	33.57	34.87
4*T*	30.40	33.13	30.87	33.15
5*T*	28.56	33.67	30.72	33.25
6*T*	30.85	34.25	31.13	34.11
7*T*	32.40	34.84	30.68	32.74
8*T*	32.34	33.29	30.85	32.25
9*T*	32.88	33.96	30.46	31.96
10*T*	32.47	34.57	29.51	33.17
11*T*	32.25	34.28	30.59	34.01
average	31.51	33.94	30.93	33.28

**Table 7:** Number of generations to obtain the convergent write strategy parameters for 3 different types of discs in every tested run of the GA with DPE_CR.

Type	1st	2nd	3rd	4th	5th	6th	7th	8th	9th	10th	Avg
1	168	135	149	156	144	126	155	129	164	166	149.2
2	177	171	159	169	155	139	161	177	156	180	164.4
3	173	162	172	167	201	145	196	180	178	171	174.5
